# A System of Human Anatomy, General and Special

**Published:** 1842-11-19

**Authors:** 


*A System of Human Anatomy, General and Special.* By ERASMUS WILSON, M. D. Lecturer on Anatomy, London. *American Edition*, Edited by PAUL B. GODDARD, A. M., M. D., &c. &c. *With one hundred and seventy illustrations by Gilbert. From the Second London Edition.* Philadelphia: Lea & Blanchard. 1843. 8vo. pp. 576.

This must prove a very acceptable book to the American medical student. It is a clear, concise and well written system of anatomy, and fully posted up to the actual state of the science. Dr. Wilson's work enjoys a high reputation in Great Britain, and from a careful examination of it we think deservedly so. It has already reached two editions, has been translated into German, and, as the author informs us, "repeated overtures have been made to Mr. Churchill for its publication in France." So perfect, indeed, is the present work, that so accomplished an anatomist as the editor, Dr. GODDARD, has been able to add but little. It has, however, undergone at his hands a careful revision, and he has occasionally appended an apt and well-written note, illustrative of the text, or vindicatory of cis-atlantic discoveries or descriptions. The illustrations are beautifully executed, and the whole book is gotten up in an admirable manner, and we trust that its sale may be commensurate with its merits and the enterprize of the publishers.

